# High-Throughput Microfluidic Electroporation (HTME): A Scalable, 384-Well Platform for Multiplexed Cell Engineering

**DOI:** 10.3390/bioengineering12080788

**Published:** 2025-07-22

**Authors:** William R. Gaillard, Jess Sustarich, Yuerong Li, David N. Carruthers, Kshitiz Gupta, Yan Liang, Rita Kuo, Stephen Tan, Sam Yoder, Paul D. Adams, Hector Garcia Martin, Nathan J. Hillson, Anup K. Singh

**Affiliations:** 1 DOE Joint BioEnergy Institute, Emeryville, CA 94608, USA; rgaillard@lbl.gov (W.R.G.); jsustarich@lbl.gov (J.S.); kzgupta@lbl.gov (K.G.); hgmartin@lbl.gov (H.G.M.); njhillson@lbl.gov (N.J.H.); 2 Sandia National Laboratories, Livermore, CA 94550, USA; 3 Biological Systems & Engineering Division, Lawrence Berkeley National Laboratory, Berkeley, CA 94720, USA; 4 Engineering Directorate, Lawrence Livermore National Laboratory, Livermore, CA 94550, USA; 5 Molecular Biophysics and Integrated Bioimaging Division, Lawrence Berkeley National Laboratory, Berkeley, CA 94720, USA; 6 Department of Bioengineering, University of California Berkeley, Berkeley, CA 94720, USA; 7 BCAM—Basque Center for Applied Mathematics, 48009 Bilbao, Spain

**Keywords:** high-throughput, electroporation, transformation, transfection, automation, microfluidic, self-driving lab, synthetic biology, strain engineering

## Abstract

Electroporation-mediated gene delivery is a cornerstone of synthetic biology, offering several advantages over other methods: higher efficiencies, broader applicability, and simpler sample preparation. Yet, electroporation protocols are often challenging to integrate into highly multiplexed workflows, owing to limitations in their scalability and tunability. These challenges ultimately increase the time and cost per transformation. As a result, rapidly screening genetic libraries, exploring combinatorial designs, or optimizing electroporation parameters requires extensive iterations, consuming large quantities of expensive custom-made DNA and cell lines or primary cells. To address these limitations, we have developed a High-Throughput Microfluidic Electroporation (HTME) platform that includes a 384-well electroporation plate (E-Plate) and control electronics capable of rapidly electroporating all wells in under a minute with individual control of each well. Fabricated using scalable and cost-effective printed-circuit-board (PCB) technology, the E-Plate significantly reduces consumable costs and reagent consumption by operating on nano to microliter volumes. Furthermore, individually addressable wells facilitate rapid exploration of large sets of experimental conditions to optimize electroporation for different cell types and plasmid concentrations/types. Use of the standard 384-well footprint makes the platform easily integrable into automated workflows, thereby enabling end-to-end automation. We demonstrate transformation of *E. coli* with pUC19 to validate the HTME’s core functionality, achieving at least a single colony forming unit in more than 99% of wells and confirming the platform’s ability to rapidly perform hundreds of electroporations with customizable conditions. This work highlights the HTME’s potential to significantly accelerate synthetic biology Design-Build-Test-Learn (DBTL) cycles by mitigating the transformation/transfection bottleneck.

## 1. Introduction

Electroporation using cuvettes has become commonplace in cell biology laboratories for delivering DNA across the cell membrane. Though frequently preferred over other approaches for commonly used bacteria such as *E. coli*, this traditional macroscale electroporation method suffers from a lack of high-throughput parallel processing capability. Additionally, electroporation of certain microbial species (e.g., gram-positive bacteria with rigid cell walls) typically results in low transformation efficiency and poor cell viability [1]. While commercial multi-well plate electroporators have attempted to address throughput limitations, they suffer from significant drawbacks including large volume requirements, lack of individual well control, and high consumable costs that limit their widespread adoption. Recently, microfluidic electroporation platforms, divided into flow-through and microelectrode array (MEA) systems, have emerged as powerful alternatives to cuvette or other macroscale technologies [2,3]. Flow-through electroporation devices process cell populations transported via pressure-driven flow through a single electroporation site. These devices are typically employed with the primary goal of generating large-scale batches of genetically modified cells [4,5]. The resulting cell population is either uniformly introduced with a common genetic sequence or subjected to a pooled library approach, which may require subsequent counterselection and sequencing. Conversely, MEAs enable parallel processing of distinct cell populations at spatially separated electroporation sites, making them well-suited for simultaneously testing multiple genetic sequences and/or cell types. Their capacity to process multiple conditions in parallel gives MEAs greater modularity and improved compatibility with conventional laboratory automation compared to flow-based devices.

The HTME platform capitalizes on these MEA advantages, with several key features of this technology underpinning its design and capabilities. Transformation efficiency is enhanced in MEAs compared to traditional cuvettes when using volumes that just fill the gaps between interdigitated electrodes, enabling optimal pore formation for enhanced molecular uptake [3,6,7]. This approach significantly improves cell viability due to the use of lower voltages and reduced sample volumes, with some studies reporting greater than 90% viability [8,9]. Much of the microscale electroporation research has focused on mammalian cells, which are highly amenable to electroporation and typically require voltages below 10 V—two to three orders of magnitude lower than bulk electroporation systems [10]. Lower voltage operation reduces Joule heating, which in turn enhances both cell viability and experimental consistency. Likewise, the increased surface to volume ratio at the microscale further mitigates Joule heating by improving heat dissipation [11]. Another feature of MEAs is their planar electrode topology, which drastically reduces reagent consumption by minimizing the electroporation volume needed to bridge electrodes compared to parallel plate electrodes in cuvettes. The scalability of MEA devices, particularly in multi-well plate formats, unlocks high-throughput experimentation by enabling parallel optimization of electroporation parameters across diverse cell types, rapid screening of genetic libraries, and exploration of complex biological phenomena.

These advantages, particularly the compatibility of the multi-well format with existing automation infrastructure, have driven the development of various MEA electroporation devices for high-throughput applications. Among the earliest examples, Huang et al. presented a microfabricated 12-well device featuring concentric circular gold-plated electrodes patterned on glass, with individual wells isolated by polydimethylsiloxane (PDMS) [8]. They reported transfection of 10 different primary cell types with transfection efficiency as high as 90%. This was achieved by spreading a thin film of cells over the electrodes to ensure all cells were exposed to the electroporation field. Building on this approach, Xu et al. presented an 18-well device featuring individually addressable interdigitated gold-plated electrodes on a PCB [12]. Improved field uniformity is achieved on PCB electrodes since they can be made much thicker than the previously described microfabricated electrodes on glass [12,13,14]. Moreover, the shift to PCB fabrication makes the device more accessible and cost effective. Unfortunately, the use of polymethyl methacrylate (PMMA) wells bonded to the PCB with PDMS and the low well density limits the device’s manufacturability and throughput, respectively. While these early devices are not high-throughput, their designs, mimicking the familiar microtiter plate format, are inherently scalable.

Rather than scaling to full microtiter plates, much of the follow-on research moved in the direction of miniaturizing the electroporation volume via the microwell format. These microwell devices are essentially miniaturized versions of microtiter plates, with compartments typically on the order of 10s to 100s of micrometers, fabricated using materials such as PDMS, polyethylene glycol, or polystyrene [15,16,17,18]. The small sizes and volumes of microwells lead to shorter diffusion distances, resulting in faster analysis times and improved sensitivity for electroporation assays. This is particularly advantageous for single-cell assays and studies focused on understanding the dynamics of the electroporation process itself [14,19]. Although microwell electroporation platforms represent a significant advancement in throughput, their manufacturing process and practical implementation is considerably more complex compared to prior approaches. Additionally, microwell architectures often lack individually addressable wells and have limited compatibility with standard laboratory instrumentation.

Driven by the need for greater compatibility with existing automation infrastructure and laboratory workflows, recent research has revisited the multi-well format for high-throughput MEA-based electroporation. For example, our group (Iwai et al.) demonstrated a high-throughput MEA electroporation device consisting of 100 wells, designed to integrate with automated liquid handlers [20]. The device was fabricated using standard microfabrication techniques, featuring an array of PDMS wells and gold-coated electrodes on a glass substrate. To ensure compatibility with conventional laboratory equipment, the device is inserted into a 3D-printed holder, providing the same form factor and well alignment as a standard 384-well microtiter plate. The work showcases the potential for MEA devices to be incorporated into high-throughput, multiplexed electroporation workflows facilitated by laboratory robots. However, the device’s wells are electrically bridged in groups of five, and the fabrication methods limit its widespread adoption. 

These existing MEA electroporation devices, while offering advantages over traditional cuvette methods, suffer from several key limitations that hinder their widespread adoption and integration into high-throughput workflows [19,21,22]. First, many devices are dependent on complex and expensive cleanroom fabrication processes, which limits their accessibility and scalability. Second, high-density formats often lack the capability for individual well addressing, restricting experimental flexibility and complicating data analysis. Furthermore, the lack of electrical isolation between wells means that a single arc event in one well can compromise the entire plate or a large group of electrically bridged wells. A third limitation is that designs are often restrictive, having been tailored for specialized applications like single-cell electroporation or biophysics studies. Finally, many MEA devices present integration difficulties due to incompatibility with standard laboratory automation equipment.

To address the limitations of existing MEA based electroporation technologies, we introduce the High-Throughput Microfluidic Electroporation (HTME) platform. The HTME operates on the first individually addressable, 384-well MEA electroporation plate (E-Plate) fabricated entirely with PCB technology in a standard microtiter plate form-factor. The use of readily available PCB fabrication technology eliminates the need for expensive and specialized cleanroom facilities, significantly reducing the cost and complexity of manufacturing. This scalable approach allows for the easy production of high-density plates, while maintaining individual well control. Additionally, individual addressing combined with PCB fabrication provides electrical isolation between wells, preventing arc events in one well from compromising adjacent wells, thus safeguarding the integrity and reliability of experiments. The standard 384-well microtiter plate format ensures seamless compatibility with existing laboratory automation equipment, facilitating end-to-end automation of electroporation workflows [23]. Finally, a key feature of the HTME platform is its precise control over the exponential-decay electroporation pulse voltage and time constant across the E-Plate, achieved through a novel emitter-follower transistor circuit that ensures consistent, user-defined electroporation pulses without requiring sample measurement. The elimination of per-well sample measurement allows for faster sequential pulsing, improving cell viability by reducing the time between electroporation and recovery.

In this work, we characterize the HTME’s performance and demonstrate its capabilities in a semi-automated workflow as a robust solution for high-throughput electroporation. Our evaluation of the E-Plate focuses on transformation efficiency and well-to-well variability, two critical metrics for high-throughput applications where consistent, reliable results across many samples are essential for effective optimization and cost-efficient operation. Using analogous electric pulse parameters, transformation efficiency in the E-Plate is 100 times lower than in cuvette-based electroporation. However, limited parameter optimization improved transformation efficiency in the E-Plate by 3-fold, demonstrating that further improvements are achievable. Our analysis indicates that the remaining gap between E-Plate and cuvette transformation efficiency is largely attributable to the excessive droplet volume per E-Plate well used in this study. According to this analysis, we believe that comprehensive parameter optimization combined with minimized droplet volumes would achieve transformation efficiencies equal to or exceeding cuvettes, consistent with the broader MEA literature. Our evaluation across multiple pulse voltage and plasmid concentration conditions shows that adjusting electroporation parameters towards values that maximize colony-forming units (CFU) significantly reduces well-to-well CFU variability. Spatial analysis of CFU distribution across two E-plates with identical conditions in all wells (varying only in dispensing order) attributes remaining well-to-well variability to liquid handling rather than to the HTME hardware itself. Future HTME system developments including E-Plate refinements, improved liquid handling, and machine learning-driven pulse optimization could enable fully automated workflows while increasing transformation efficiency and reducing variability across diverse cell types.

## 2. Materials and Methods

### 2.1. E-Plate Design

**Figure 1:**
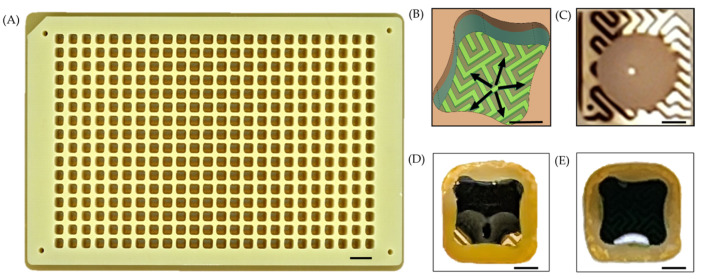
(**A**) Photograph of an E-Plate. (**B**) Rendering of the interdigitated electrode geometry illustrating the outward capillary flow. (**C**) A 2 μL droplet of *E. coli* beads on the well surface without a surface tension trap. (**D**) A 2 μL droplet of *E. coli* in a well with a surface tension trap exhibits nearly complete wetting. (**E**) A 3 μL droplet of *E. coli* demonstrates complete wetting within the surface tension trap. Note that food dye was added to the cells to improve image contrast and visualization in (**D**,**E**). Note, the scale bar on A represents 10 mm and the scale bars on (**B**–**E**) represent 1 mm.

A flexible printed circuit (FPC) board serves as the substrate for the E-Plate (Figure 1A). The top surface of the FPC supports an array of gold-plated interdigitated electrodes, which are fabricated with a ½ oz copper pour and arranged in the layout of a standard 384-well microtiter plate. The FPC layer is bonded to precision-milled FR4 wells using pressure-sensitive adhesive (PSA) in a heat press. The wells themselves have a profile of 3.4 × 3.4 mm and a height of 6 mm, providing a maximum working volume of 65 μL. A total of 388 contact pads are present on the underside of the FPC, enabling electrical connection to the HTME. Each of the 384 wells has a dedicated contact pad, while the remaining 4 contact pads serve as shared ground connections to ensure a stable and uniform electrical reference across all wells.

The FPC comprises three copper layers serving as ground planes. These layers not only shield the electroporation pulse from coupling between traces within the FPC but also enhance the structural rigidity and planarity of the thin substrate. Furthermore, the increased heat capacity provided by the copper layers contributes to uniform thermal control of the droplets within the wells. 

Each electrode-pair exhibits a 100 µm trace-width, 100 µm inter-electrode gap, and a 35 µm profile height. The interdigitated design leverages capillary action to induce an omni-directional flow of the aqueous electroporation sample, drawing it from the center towards the well’s periphery (Figure 1B). Consequently, the contact area between the sample and electrodes is increased, enabling the electric field to permeate a greater volume of the sample. 

Thin FR4 protrusions present at the bottom of each well act as surface tension traps. These traps prevent droplets from clinging to the well walls and, in conjunction with the interdigitated electrode design, ensure efficient and even spreading of the sample across the electrodes. A minimum droplet volume of 3 μL reliably fills these traps, creating a consistent and uniform volume fraction of the sample exposed to the electric field across all wells (Figure 1C–E). This consistent electrode coverage enhances reproducibility across the plate. The trap’s size and shape can be modified to adjust the minimum droplet volume required to completely wet the electrodes.

### 2.2. HTME Design

**Figure 2:**
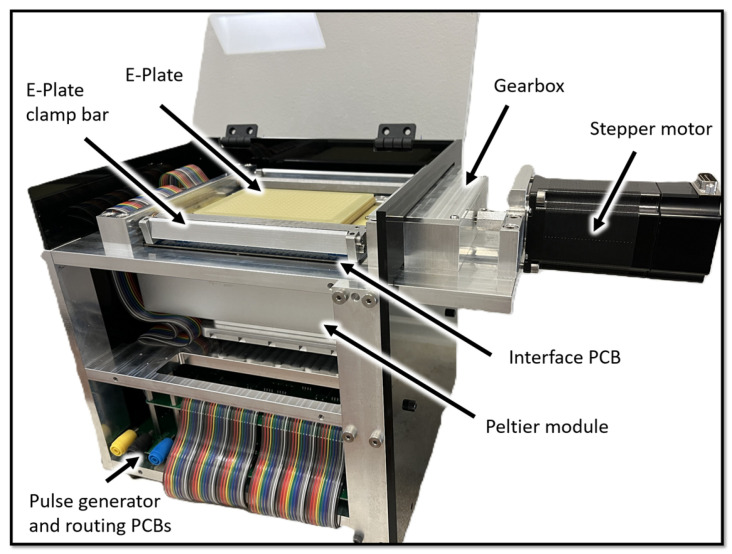
The HTME housing, containing all the mechanical and electrical components of the device. Key components are labeled, including the stepper motor, gearbox, spring loaded E-Plate clamping mechanism, Opentrons Peltier module (Long Island City, NY, USA), pulse generator and routing PCBs, E-Plate, and interface PCB.

A custom CNC-milled aluminum frame securely contains all the mechanical and electrical components of the HTME control-unit (Figure 2). The top of the housing features a receptacle that integrates several key elements: spring-loaded pogo-pins that relay the electroporation voltage pulse to the E-Plate, guides for precise E-Plate positioning, and a motorized clamping mechanism. When engaged, the clamping mechanism presses the electrical pads located on the underside of the E-Plate onto the pogo pins in the interface board (described in the following section), providing a secure electrical connection from the control electronics to the E-Plate’s wells. To overcome the cumulative 74 lbs of spring-force produced by the 388 pogo pins, a custom-built 4.34:1 gearbox amplifies the torque from the stepper motor providing more than 90 lbs of force in the spring-loaded clamping mechanism. This stepper motor (C0-5718L-01P, Lin Engineering, Morgan Hill, CA, USA) interfaces directly with a computer via USB and is controlled using the manufacturer’s software.

The pogo pins are soldered to an interface PCB, which serves as a central hub for electrical connections between the E-Plate and the pulse routing and pulse generation PCBs. An aluminum heat transfer block located in the cutout in the interface PCB, is positioned between the E-Plate and an Opentrons Temperature Module. This module contains a Peltier element and sits on a spring-loaded base plate, which the clamping mechanism compresses to maintain firm contact with the underside of the E-Plate, maximizing heat transfer to the wells.

To make efficient use of space, pulse routing is handled by two PCBs situated below the Opentrons Temperature Module. The top pulse routing board is populated with 192 solid-state relays (SSRs), while the bottom routing board contains another set of 192 SSRs along with the pulse generator itself. Together, these 384 SSRs are responsible for selectively routing the electroporation pulse to the appropriate wells on the E-Plate via the interface board. Normally, wells are pulsed individually, but simultaneous pulsing of multiple wells is also possible. The high-voltage power supply currently exists as a standalone breadboard unit but will be integrated onto a dedicated PCB within the housing in future iterations of the HTME.

### 2.3. Electronic Control and Pulse Generation

**Figure 3:**
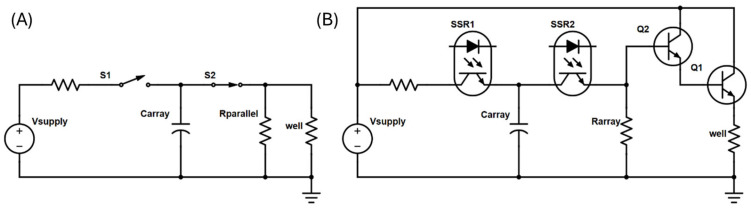
Representative exponential pulse generator schematics for (**A**) typical cuvette electroporators and (**B**) the HTME.

Traditional electroporator pulse generators employ a resistor-capacitor (RC) circuit to generate exponential decay pulses (Figure 3A). The pulse time constant is determined by the selected capacitor(s), the cuvette impedance, and the additional resistor(s) placed in parallel with the cuvette. The added parallel resistance also improves the reliability of the delivered pulse time constant. While some pulse generators require manual RC value selection to achieve a desired time constant, necessitating user trial and error, others offer automated control by measuring cuvette impedance and adjusting RC values accordingly. However, such impedance-based probing mechanisms are impractical for the HTME due to the challenges of accurate and rapid measurement of the impedance of microliter droplets.

To overcome this challenge, the HTME employs a fundamentally unique pulse generator circuit for electroporation based on an NPN BJT Darlington pair in an emitter follower configuration (Figure 3B). This design eliminates the need for well impedance measurements by directly controlling the pulse time constant through the Darlington pair. The process involves charging a capacitor array and then subsequently discharging it across a resistor array connected in parallel to the Darlington pair base, generating a precise exponential decay pulse at the emitter, which is then delivered to an E-Plate well via the routing PCBs. This approach ensures consistent pulse characteristics across all wells, regardless of variations in droplet impedance, simplifying operation and enhancing experimental reproducibility.

An onboard sensor, validated against a commercial oscilloscope, measures the pulse voltage at the Darlington pair emitter before routing to the E-Plate well electrode, providing feedback on the delivered voltage. Pulse voltage is controlled by a custom, linearly adjustable power supply (0–360 V). The HTME generates exponential decay pulses using a resistor array (0.5, 1, 2, 2, 5, 10, and 20 kΩ) and a capacitor array (10, 20, 20, 50, 100, 200, 200, 500, and 1000 nF), achieving time constants ranging up to 85 ms.

While the specific capacitance and resistance values used in traditional pulse generators are often reported in the literature, these values are not directly comparable to those used in the HTME due to the fundamental differences in pulse generation mechanisms. The resulting time constant is a more meaningful parameter for comparing pulse characteristics between the HTME and other electroporation systems. However, even when comparing time constants, a potentially significant distinction arises. The HTME delivers the full power of the pulse directly to the well, unlike traditional electroporators utilizing a parallel resistor to adjust the time constant, diverting an unknown portion of the electrical power away from the sample. This difference in power delivery may impact transformation efficiency and warrants further investigation [24].

The HTME is operated using a graphical user interface (GUI) built using Matlab App Designer version R2019a. The GUI communicates with the HTME onboard microcontroller via a USB connection to a PC, enabling users to define and execute customized electroporation protocols for each well on the E-Plate. The GUI allows the user to control pulse parameters and select from different pulsing orders such as row-by-row, column-by-column, randomized, and optimized “fast” mode for minimizing total pulse time. Detailed pulse data, including measured voltage, time constant, and well pulse order, is automatically logged for analysis. A debugging feature provides real-time error reporting and voltage plots for each well during electroporation, enabling experimental monitoring and troubleshooting. Any number and combination of wells may be populated and electroporated or left empty.

### 2.4. Strains, Materials, and Reagents

The *E. coli* DH10β strain and pUC19 plasmid were obtained from the Joint BioEnergy Institute Public Registry (https://registry.jbei.org/folders/2604, accessed on 1 June 2025). The pUC19 plasmid (JBEI-003120, JPUB_026532) harbors a β-lactamase gene for resistance to various lactam antibiotics (e.g., ampicillin or carbenicillin) for selection. A single colony of *E. coli* DH10β (JBEI-003596, JPUB_026535) from an LB agar plate was inoculated into 5 mL LB and grown overnight. Following overnight growth, 2 mL of culture was inoculated into 200 mL of fresh LB in a 1 L Erlenmeyer flask and incubated at 37 °C at 200 RPM until the culture reached an optical density (OD600) of 0.4. The cells were made electrocompetent by first chilling on ice for 20 min, followed by centrifuging at 3000 RPM at 4 °C for 20 min. The supernatant was decanted, and the cells were washed and centrifuged three times with ice-cold deionized water. Finally, the cells were washed and centrifuged as before with ice-cold 10% glycerol, then resuspended, aliquoted, and stored at −80 °C until use. 

Deep-well plates (RAININ, Oakland, CA, USA) were used for post-electroporation recovery, and 48-well Bioassay QTrays (Molecular Devices, San Jose, CA, USA) were used for plating the recovered cells. Reagents for LB and super optimal broth with catabolite repression (SOC) were sourced from Sigma-Aldrich (St. Louis, MO, USA). Prior to each run on the HTME, E-Plates were sterilized by soaking in 99% isopropanol for 3–5 min, followed by a soak in 75% ethanol for 3–5 min. After the alcohol was removed, plates were rinsed with Milli-Q water at least three times or until no residue remained. E-Plates were then air-dried in a 50 °C oven for 1–2 h or at room temperature overnight. Finally, E-Plates were UV-treated for 30–45 s using a Spectrolinker™ XL-1000 Series UV Crosslinker and sealed with aluminum foil (Microseal Foil Seals, Bio-Rad, Hercules, CA, USA) until use.

### 2.5. Electroporation Protocol

Aliquots of DH10β *E. coli* electrocompetent cells stored at −80 °C were thawed on ice for 20 min. Cells were premixed with pUC19 to minimize covariance and maximize uniform contact between cells and plasmid. When testing different plasmid concentrations, separate premixed stocks were prepared for each concentration. For more complex experimental designs involving plasmid and strain libraries, users may dispense reagents separately into the E-Plate. A Formulatrix Mantis liquid handler was used to dispense 3 μL droplets of premixed *E. coli* electrocompetent cells and pUC19 plasmid into the E-Plate wells. All materials and reagents were kept on ice before electroporation.

After dispensing, the E-Plate was sealed with an AeraSeal gas permeable membrane (Excel Scientific, Victorville, CA, USA) and then transferred to the HTME E-Plate receptacle for electroporation. After electroporation, 40 μL of SOC recovery medium was promptly added to the E-Plate wells using a Hamilton Vantage liquid handler. After mixing, 10 μL of the cell recovery mixture was transferred from the E-Plate to deep well plates containing 990 μL of SOC for incubation (shaken at 200 rpm at 37 °C for 1 h). 100 μL of the cell recovery mixture was plated on the QTrays using the Hamilton Vantage handler, dried in WhisperFlow Cabinet for 30 min, and subsequently incubated overnight at 37 °C. CFU on QTrays were imaged using a Molecular Devices QPix Microbial Colony Picker, and ImageJ version 1.54p (Supplementary Material) was used to analyze the images and obtain CFU counts.

A control was established using a set of 3 cuvettes with a 1 mm electrode gap. Cells were premixed with 0.02 ng/μL pUC19. After 15 min of incubation on ice, 50 μL of cells were electroporated with a 1800 V and 5 ms time constant pulse. Cells from each cuvette were recovered in 1 mL of SOC medium, transferred to 1.5 mL Eppendorf tubes, and incubated as before. After a 10,000× dilution with water, 200 μL of diluted recovery was plated on LB agar, incubated overnight at 37 °C, imaged, and analyzed as above.

## 3. Results

### 3.1. Transformation Efficiency, Variability, and Electric Field Dynamics

**Figure 4:**
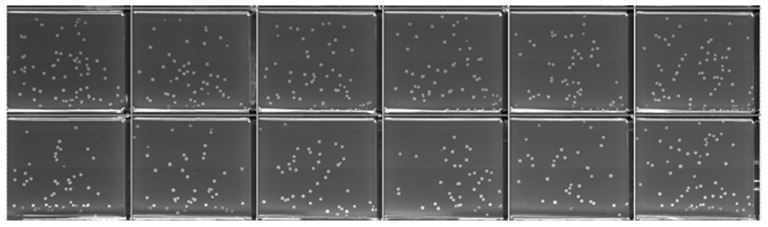
Image of *E. coli* colonies expressing the selectable marker on a QTray (inner well dimensions: 3.0 × 2.5 cm) following successful transformation using the HTME platform. All wells were subjected to identical conditions with pulse parameters of 180 V and 5 ms time constant, using 0.02 ng/μL pUC19 plasmid. Note, a fraction of the recovered cells were plated to facilitate colony counting and prevent lawn formation (Section 2.5). The image is cropped from a QPix-generated composite of individually imaged wells. The full QTray image is available in the Supplementary Material.

This study evaluates the performance of the HTME for high-throughput electroporation by comparing the E-Plate performance to cuvette under analogous conditions. Exponential decay pulses with targeted parameters of 180 V peak voltage and 5 ms time constant were applied uniformly across an E-Plate containing *E. coli* premixed with 0.02 ng/μL of pUC19 plasmid using the procedure described in Section 2.5. This voltage target was scaled down from 1800 V recommended by BioRad’s *E. coli* Pulser Transformation Apparatus Operating Instructions and Applications Guide for a 1 mm gap cuvette to accommodate the E-Plate’s 100 µm electrode gap. The HTME onboard sensor confirmed precise pulse delivery across the plate, with measured values of 179 V ± 1 V peak voltage and 5.0 ms ± 0.1 ms time constant. The system pulsed wells sequentially—left-to-right across each row, then advanced row-by-row downward—completing all 384 pulses in 48 s. Successful transformants were observed in 382 wells, confirming the E-Plate’s effectiveness under the applied conditions. Notably, no electrical arcing occurred during the procedure, and all QTrays remained free of contamination. Figure 4 shows representative CFU cropped from a 48-well QTray composite image to better visualize individual colonies.

An average of 48 CFU per well was observed across the E-Plate, yielding a coefficient of variation (CV) of 35%. This level of variability is acceptable for demonstrating platform functionality and for applications with efficient transformations like *E. coli*. However, for difficult-to-transform organisms that often yield zero or few CFU, minimizing variability becomes crucial to distinguish successful optimization from random variation and to identify parameters that minimize cell and DNA requirements while ensuring reliable CFU production across wells. Significant contributors to the high CV include technical factors that can be addressed, such as non-optimized electroporation parameters and liquid handling protocols that require refinement for the multi-well plate format. Our experiments presented in Section 3.2 address these technical factors through an investigation of pulse voltage and plasmid concentration parameters alongside a statistical analysis of dispensing patterns, achieving a substantially improved CV of 15%. These results demonstrate several of the platform’s key advantages, including dramatically reduced reagent consumption per transformation, seamless automation integration, and the ability for rapid transformation across hundreds of wells.

The average transformation efficiency for three cuvette controls and the E-Plate was calculated to be 3.395 × 10^9^ and 3.477 × 10^7^ CFU/μg, respectively. Despite reports that MEA-based systems typically outperform cuvettes in transformation efficiency, the E-Plate exhibited significantly lower efficiency. We attribute this discrepancy to two primary factors. The first one is the use of large cell volumes. MEAs generate a non-uniform electric field that decays exponentially from the electrode surface into the cell suspension above, limiting field penetration compared to the uniform field produced by the cuvette’s parallel plate electrodes. Even so, MEAs generally achieve higher efficiencies by utilizing dilute cell suspensions at volumes just sufficient to fill the gap between interdigitated electrodes, ensuring uniform electric field exposure. This approach enables MEAs to achieve a field distribution similar to that of cuvettes while reducing the impact of Joule heating, resulting primarily from lower operating voltages that reduce current and consequently resistive heating, with the increased surface-to-volume ratio further enhancing heat dissipation. These combined effects improve cell viability and transformation efficiency. In contrast, the E-Plate was designed to accommodate dense cell suspensions that bead rather than spread across the electrodes. We use dense cell suspensions for this study because they are more common in routine microbial laboratory work and beneficial for electroporating difficult-to-transform organisms. 

**Figure 5:**
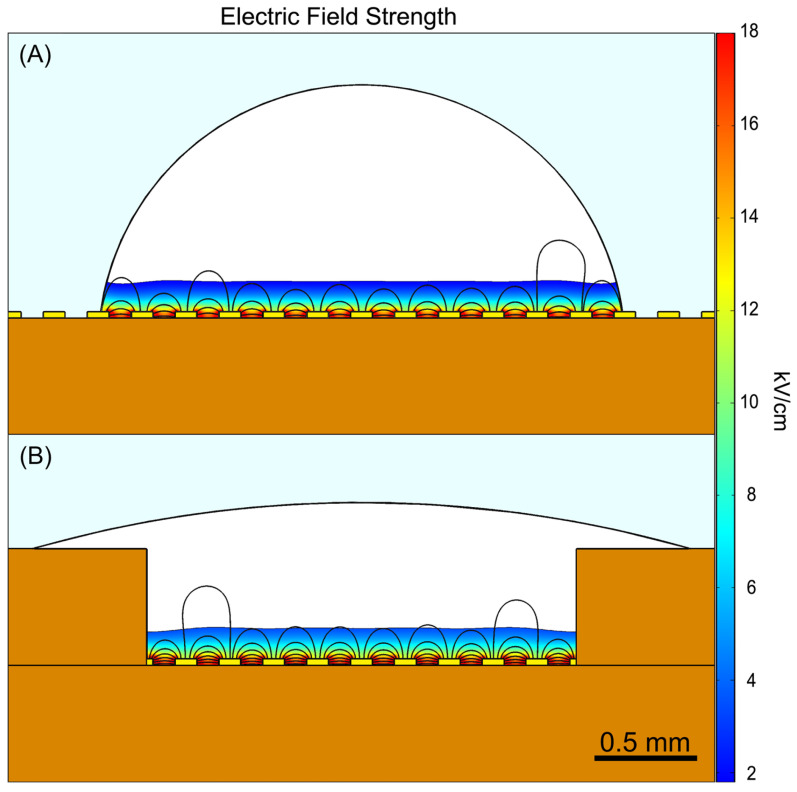
Electric field distribution within 3.0 µL droplets with an applied voltage of 180 V shown as 2D cross-sectional views simulated using COMSOL Multiphysics V5.5. (**A**) A beaded hemispherical droplet and (**B**) a droplet in the surface tension trap. Both droplets have approximately 25% of their volume exposed to >10% of the maximum field strength of 18 kV/cm (colored region). The trap ensures consistent electrode coverage across wells. Future iterations of the trap geometry could enable lower droplet volumes achieving higher transformation efficiency while maintaining uniform droplet/electrode coverage. The color scale represents electric field strength in kV/cm. Note, this simulation utilizes water, with a relative permittivity of 80 and conductivity of 5.6 × 10^−4^ [S/m], as a representative model for illustrative purposes and does not account for the complex dielectric properties of cells and DNA.

However, these dense suspensions form droplets where the majority of the volume is distant from the electrode surface, as illustrated in Figure 5A. While a hydrophilic coating could improve wetting, a more scalable and mass-manufacturable solution was implemented in the form of a surface tension trap to induce droplet spreading (Figure 5B), as detailed in Section 2.1. As shown in Figure 5A, when a 3 µL droplet adopts a beaded hemispherical shape, approximately only 25% of its volume is exposed to >10% of the maximum electric field strength (shown as the colored region). The surface tension trap maintains a similar volume fraction exposed to effective field strengths but provides two critical advantages for reliable electroporation. First, it ensures consistent electrode coverage across all wells, whereas droplets without the trap exhibit variable spreading patterns that can lead to disparate transformation outcomes despite otherwise identical conditions. Second, the trap design provides a foundation for future optimization: modified trap geometries could enable the use of smaller volumes while maintaining low well-to-well variability, and could potentially be engineered to increase the volume fraction exposed to transformation-sufficient field strengths, thereby improving overall efficiency.

While larger electroporation volumes result in a lower fraction of cells exposed to sufficient electric field strength for transformation, and therefore lower overall transformation efficiency, the use of large droplets was necessary for our experimental design. As described in Section 2.1, smaller volumes do not consistently wet the electrodes, which was problematic for two reasons. First, uniform wetting across all wells was essential for accurate assessment of the E-Plate’s performance, enabling us to determine whether transformation outcomes potentially varied systematically based on well position within the plate due to electronic factors. Second, inconsistent electrode coverage would introduce an additional source of variation that would confound our ability to isolate and assess other sources of variability. Although this approach sacrificed maximum efficiency, it established a reliable foundation for validating the E-Plate functionality. Future studies with improved E-Plate well design can explore reduced volumes, increasing transformation efficiency while maintaining acceptable consistency for applications requiring minimal CV.

The second factor contributing to lower transformation efficiency in the E-Plate is the use of pulse parameters recommended for cuvettes rather than for the E-Plate’s unique properties. The smaller working volume between electrodes in the E-Plate results in lower sample resistance, potentially requiring pulse parameter adjustments. Moreover, the E-Plate’s smaller sample volume and lower voltage operation reduce Joule heating, which permits the use of higher field strengths that enable larger and more numerous pore formation when properly optimized.

### 3.2. Improving Efficiency and Reducing Variability

**Figure 6:**
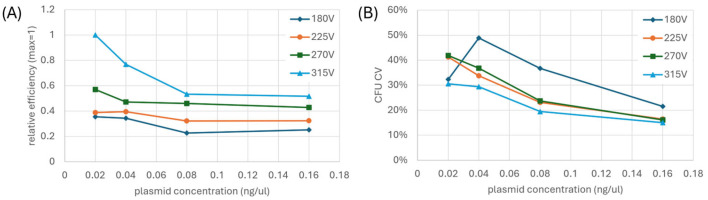
(**A**) Normalized transformation efficiency from combined experimental runs indicates that transformation efficiency increases with increasing voltage and (**B**) normalized CV in CFU from combined experimental runs indicate that CFU variability decreases with increasing pulse voltage and plasmid concentration. 24 replicates were used per condition with a couple exceptions. A dispensing error occurred in the second run that caused all droplets for the 225 V, 0.16 ng/μL and half of the droplets for the 180 V, 0.16 ng/μL conditions to partially fill the E-Plate. As a result, the data for the 225 V, 0.16 ng/μL condition could not be combined and normalized. Instead, we employed a proportional ratio-based estimation approach to calculate the relative efficiency and CV for this condition as described in the Supplementary Material.

To address these electrode geometry differences, we investigated the effects of pulse voltage and plasmid concentration on transformation efficiency in the E-Plate. We tested four pulse voltages (180, 225, 270, and 315 V) against four plasmid concentrations (0.02, 0.04, 0.08, and 0.16 ng/μL), with each condition replicated 24 times per E-Plate and repeated on a second E-Plate. Figure 6A shows the combined normalized data from both experiments. We present transformation efficiency as normalized relative values because absolute efficiency varies significantly between experimental runs due to numerous factors such as cell preparation and liquid handling, making relative improvements more meaningful for guiding experimental design. Notably, the 315 V condition consistently delivered 2–3 fold higher efficiency than the 180 V condition, indicating that the interdigitated electrode configuration requires higher field strengths than cuvette for optimal transformation. We observed an apparent stabilization of transformation efficiency at higher plasmid concentrations (0.08–0.16 ng/μL), which may result from a biological threshold effect specific to the non-uniform field distribution of our electrode geometry. Specifically, in regions where field strength is just at the threshold for permeabilization, higher plasmid concentration may compensate for marginally effective pore formation. These results demonstrate that the unique electrical characteristics of the E-Plate necessitate different operating parameters compared to conventional cuvette systems, particularly regarding optimal electric field strength.

Interestingly, one well in one E-Plate replicate experienced electrical arcing yet still produced CFU. This phenomenon has been observed occasionally across various E-Plates. We attribute this to the large sample volume in E-Plates, where the arc event may kill cells near the electrodes while generating fields of sufficient strength to electroporate cells in regions normally unexposed to transformation-adequate field strengths. Notably, a key feature of the individual well addressability design is that arc events remain isolated to individual wells, allowing experiments to continue unaffected in all other wells. CFU data from this arced well was not included in our analysis.

We further analyzed the reproducibility of transformation by examining the CV in CFU across the different experimental conditions. As shown in Figure 6B, we generally observe that CV decreases with both increasing voltage and increasing plasmid concentration. The inconsistencies in the overall trends reflect substantial differences between our two experimental replicates. In one experimental replicate, nearly half of the tested conditions exhibited variability at or above 40%, whereas in the other replicate, only a single condition reached 40% variability and none exceeded this threshold. Even so, all voltage conditions converged toward similar CV values at the highest plasmid concentration (0.16 ng/μL), suggesting factors beyond optimization of electroporation parameters may limit reproducibility. As we demonstrate later in this section, the remaining variability appears to be largely attributable to liquid handling effects, emphasizing the need for E-Plate-specific liquid handling optimization, where reproducibility is required.

Future optimization of electroporation pulse parameters will require varying voltage and plasmid concentration in finer increments and across extended ranges to maximize transformation efficiency, particularly for challenging organisms, and to minimize reagent consumption in high-throughput workflows. This comprehensive parameter mapping, however, is only feasible when experimental variability is minimized, making reproducibility a critical prerequisite for efficient protocol development. Statistical power analysis demonstrates that decreasing CV from 40% to 10% dramatically reduces the required replicates from 63 to just 4 for detecting a 20% effect size—a nearly 16-fold improvement in experimental efficiency. To put this in practical terms, discriminating between 96 different conditions with a ≥20% effect size would require 16 E-Plates (6048 wells total) when variability is high (40% CV), while the same experiment with low variability (10% CV) could be completed on a single E-Plate. This order-of-magnitude difference in resource requirements fundamentally changes what experiments become feasible. Moreover, low variability is particularly useful for developing robust machine learning models for electroporation optimization, as high-variance data compromises model training and predictive accuracy. As we expand our protocol exploration to include additional parameters such as pulse duration, cell density, and other key variables, maintaining low experimental variability will be essential for efficiently identifying truly optimal conditions rather than artifacts of experimental noise.

Beyond parameter optimization, improving liquid handling precision offers another significant avenue to reduce transformation variability. Spatial analysis of CFU across the E-Plate from the experiment in Section 3.1 revealed non-random clustering of high and low performers. When we altered only the dispensing pattern from column-wise to row-wise while keeping all other variables constant, these spatial clusters completely reorganized, indicating clustering is not attributable to the E-Plate or HTME. Local Indicators of Spatial Association (LISA) analysis confirmed clustering in both experiments (Moran’s I = 0.28 for column-wise and 0.15 for row-wise dispensing, where values range from −1 to +1; positive values indicate spatial clustering of similar CFU counts, negative values indicate dispersion, and 0 represents random distribution). The LISA methodology and cluster maps are provided in the Supplementary Material. To quantify the degree to which spatial patterns persisted across dispensing methods, we calculated pattern similarity between experiments, finding minimal pattern retention (normalized pattern similarity = 2%, where 0% represents random overlap and 100% represents identical patterns). ANOVA further validated the dispensing-dependent spatial effects. With column-wise dispensing, both column effects (F = 7.5, *p* < 10^−20^) and row effects (F = 3.2, *p* < 10^−5^) became significant; however, with row-wise dispensing, only row effects were significant (F = 4.0, *p* < 10^−6^) while column effects were not (F = 1.1, *p* = 0.35). This statistical evidence strongly supports liquid handling as the primary driver of position effects on CFU. 

Though both liquid handlers have a manufacturer-specified dispense error of <3%, the clustered distribution of transformation efficiencies suggests intermittent, non-random perturbations during liquid handling. These may be due to thermal heterogeneity or transient, localized, variations in cell density due to cell settling in the dispensing nozzle. While operating at higher voltages and plasmid concentrations reduces overall CV, a significant portion of the remaining variability likely stems from dispensing inconsistencies or other compounding liquid handling effects. This suggests that even at optimal electroporation parameters, addressing liquid handling variability remains crucial for maximizing experimental throughput. Recognizing this challenge, we have initiated efforts to improve dispensing precision by exploring various protocol modifications including investigating different dispensing head types, varying cell density, improving temperature control during dispensing, and testing additional dispensing technologies and platforms. In the meantime, randomizing condition positions may provide an effective strategy to prevent position-dependent effects from biasing specific test conditions, ensuring more reliable data while continued improvements in liquid handling are pursued.

## 4. Discussion

This work demonstrates the proof-of-concept functionality of the HTME platform, with successful transformation of *E. coli* and pUC19 in the novel electroporation well plate, or E-Plate. Although this study focuses on *E. coli*, the HTME platform’s versatility is underscored by the extensive literature demonstrating successful transformations of diverse cell types using analogous electrode designs. The HTME platform exhibits consistent well-to-well variation under uniform conditions. This consistency, coupled with the platform’s inherent advantages of well-to-well experimental flexibility and compatibility with laboratory automation, positions the HTME as a powerful tool for accelerating synthetic biology workflows.

The true potential of the HTME platform lies in its capacity to rapidly explore large experimental spaces, fundamentally transforming how synthetic biology research is conducted. One key area of impact will be the rapid optimization of electroporation parameters. The principles of electroporation are generally applicable across different cell types and cargo, but in some cases, the parameters and strategies can be highly complex and difficult to optimize. The ability to test a wide range of experimental conditions in parallel—from electric field parameters to electroporation buffers and cell preparation protocols—will be invaluable for optimizing challenging transformations where optimal parameters are not defined [25].

Beyond addressing difficult-to-transform organisms, the HTME platform enables significant resource optimization. The system’s microliter working volumes dramatically reduce consumption of valuable reagents, such as custom-synthesized DNA and precious primary cells, compared to traditional cuvette methods. By simultaneously testing numerous parameter combinations, researchers can rapidly identify conditions that maximize transformation efficiency while minimizing input requirements—a crucial advantage when working with limited or expensive biological materials. This combination of optimized protocols and reduced resource requirements makes previously cost-prohibitive experiments feasible, expanding the scope of practical synthetic biology applications.

Enabled by these optimization capabilities, the HTME platform potentially enables efficient exploration of large genetic landscapes using multiplex automated genome engineering (MAGE) or CRISPR-based approaches [17,20,23,26,27]. By enabling the parallel transformation of hundreds of biological constructs, the HTME streamlines library-based workflows. Applications include screening pre-existing libraries for rare, high-performing candidates, uncovering synergistic interactions within combinatorial assemblies of genetic parts, and accelerating directed evolution experiments through rapid cycles of genetic edits, transformation, and screening.

The HTME platform’s compatibility with automation makes it an ideal candidate for integration into fully automated self-driving labs (SDLs) [28,29]. The E-Plate’s standardized microtiter plate format allows for seamless integration with liquid handling robots and other automated equipment, permitting the flexible configuration of pipelines requiring high-throughput electroporation. In this context, the HTME platform has the potential to dramatically accelerate the Design-Build-Test-Learn (DBTL) cycle in synthetic biology, by eliminating the electroporation bottleneck and permitting the collection of large amounts of data that can train machine learning algorithms to make more effective recommendations for the next cycle. This enhanced performance of the Learn phase of the DBTL cycle, will ultimately expedite the discovery and optimization of novel biological solutions to global challenges.

While this vision of a fully automated synthetic biology future is highly compelling, it’s important to note that the E-Plate and HTME technology is still in its early stages. The work presented here provides a critical first step by demonstrating the feasibility of this platform. The successful transformation of *E. coli* with pUC19 using the E-Plate and the HTME system validates its core functionality and highlights its potential for broader applications. However, significant opportunities for improvement and expansion remain.

Continued HTME development will focus on achieving comparable or superior transformation efficiency to traditional cuvettes by optimizing the electric field and reducing the working volume in each well. To optimize electroporation for a wider range of cell types and applications, the system will be upgraded to generate additional pulse shapes, including square and bipolar pulses, which improve transformation efficiency and reduce cell death compared to traditional exponential decay pulses in some organisms [24,30,31]. We foresee expanding to higher density E-Plates and integrating additional downstream processing steps, such as on-plate cell culture, incubation, and analysis, to further streamline and miniaturize the entire synthetic biology workflow. By merging these capabilities onto a single, automated platform, we can empower researchers to explore increasingly complex biological questions with previously unattainable scale and efficiency.

## 5. Conclusions

This work introduces a powerful new tool for the synthetic biology community—a PCB-fabricated, individually addressable, 384-well electroporation plate that significantly advances high-throughput electroporation by substantially reducing reagent consumption, enabling protocol optimization, and seamlessly integrating with automated workflows. The HTME platform’s novel pulse generation mechanism provides customized, rapid, and precise pulse delivery without sample measurement. Our validation experiments with *E. coli* establish a foundation for further refinement while confirming the E-Plate’s viability for high-throughput applications. The HTME enables new research possibilities through its capacity to simultaneously test diverse experimental conditions, dramatically accelerating optimization for challenging transformations. By integrating seamlessly with laboratory automation systems, the HTME platform empowers researchers to accelerate the DBTL cycle in synthetic biology and enable the discovery and optimization of novel biomolecules, pathways, and genetic circuits. As SDLs become increasingly prevalent and synthetic biology pushes the boundaries of our ability to engineer living systems, platforms like the HTME will be essential for bridging the gap between ambitious design and efficient experimental realization.

## Data Availability

The raw data supporting the conclusions of this article will be made available by the authors upon request.

## Supplementary Materials

The following supporting information can be downloaded at: https://www.mdpi.com/article/10.3390/bioengineering12080788/s1, Figure S1: LISA cluster maps comparing column-wise (top) and row-wise (bottom) dispensing methods, Figure S2: Theoretical LISA cluster maps demonstrating extreme spatial autocorrelation patterns.

## Abbreviations

The following abbreviations are used in this manuscript:

DNA	Deoxyribonucleic Acid
HTME	High-Throughput Microfluidic Electroporation
E-Plate	electroporation plate
PCB	printed-circuit-board
*E. coli*	*Escherichia coli*
DBTL	design-build-test-learn
MEA	microelectrode array
PDMS	polydimethylsiloxane
PMMA	polymethyl methacrylate
3D	three dimensional
CFU	colony-forming units
FPC	flexible printed circuit
PSA	pressure-sensitive adhesive
CNC	computer numerical control
USB	universal serial bus
SSR	solid-state relay
RC	resistor-capacitor
NPN	negative-positive-negative
BJT	bipolar junction transistor
GUI	graphical user interface
PC	personal computer
LB	Luria-Bertani
SOC	super optimal medium with catabolic repressor
UV	ultraviolet
CV	coefficient of variation
LISA	local indicators of spatial association
ANOVA	analysis of variance
MAGE	multiplex automated genome engineering
CRISPR	clustered regularly interspaced short palindromic repeats
SDL	self-driving lab
