# Supplementary material for: High-Throughput Microfluidic Electroporation (HTME): A Scalable, 384-Well Platform for Multiplexed Cell Engineering
Source: Bioengineering (Basel). 2025 Jul 22;12(8):788. doi: 10.3390/bioengineering12080788 (PMC12383916; doi:10.3390/bioengineering12080788)
Supplement: Supplementary file 1 [file bioengineering-12-00788-s001.zip › bioengineering-3721094-supplementary.pdf]

Supplementary Information

High-Throughput Microfluidic Electroporation (HTME): A Scalable, 384-Well Platform for Multiplexed Cell Engineering

William R. Gaillard[1,2,4], Jess Sustarich[1,2], Yuerong Li[1,2,3], David N. Carruthers[1,4], Kshitiz Gupta[1,3], Yan Liang[1,4], Rita Kuo[1,4], Stephen Tan[1,4], Sam Yoder[1,4], Paul D. Adams[1,5,6], Hector Garcia Martin[1,4,7], Nathan J. Hillson[1,4], and Anup K. Singh[1,2,3,*]

1 DOE Joint BioEnergy Institute, Emeryville, CA 94608, USA
2 Sandia National Laboratories, Livermore, CA 94550, USA
3 Engineering Directorate, Lawrence Livermore National Laboratory, Livermore, CA 94550, USA
4 Biological Systems & Engineering Division, Lawrence Berkeley National Laboratory, Berkeley, CA 94720, USA
5 Molecular Biophysics and Integrated Bioimaging Division, Lawrence Berkeley National Laboratory, Berkeley, CA 94720, USA
6 Department of Bioengineering, University of California Berkeley, Berkeley, CA 94720, USA
7 BCAM, Basque Center for Applied Mathematics, Bilbao 48009, Spain

* Correspondence: singh46@llnl.gov

ImageJ script used for counting colony-forming units

This ImageJ macro enables batch colony counting for .tiff images captured by the Molecular Devices QPix Microbial Colony Picker. The macro first prompts the user to select an input folder containing all the .tiff images and an output folder. Users can adjust the colony size, circularity, range, and define a region of interest to exclude overexposed areas near the QTray borders to prevent miscounting. Once initiated, the macro processes all images and saves them with an overlay showing the detected colonies, parameters (such as major and minor axes) of the fitted circle for all colonies detected in the "Results.txt" file, and a summary of number of colonies detected, average size, and circle fitting parameters for each image in the "Summary.txt" file.

```
macro "CFU count"{
//Initially prompted with two windows; first is input images and second is output images with overlay (light blue)
Dialog.create("Input")
Dialog.addMessage("Select an Input Folder")
Dialog.show();
Idir = getDirectory("Choose Input Directory");
Dialog.create("Output")
Dialog.addMessage("Select an Output Folder")
Dialog.show();
Odir = getDirectory("Choose Output Directory");
Dialog.create("Image Specifications")
//Change size and circularity to adjust for differences between colonies
Dialog.addNumber("Max Circularity:", 1.00);
Dialog.addNumber("Min Circularity:", 0.750);
Dialog.addNumber("Max Size:", 0.01);
Dialog.addNumber("Min Size:", 0.0004);
```

```
//Use x, y, xWidth, yWidth to crop out region of interest or remove overexposed borders
Dialog.addNumber("x", 0);
Dialog.addNumber("y", 30);
Dialog.addNumber("xWidth", 700);
Dialog.addNumber("yWidth", 655);
Dialog.show();
max_circ=Dialog.getNumber();
min_circ=Dialog.getNumber();
max_size=Dialog.getNumber();
min_size=Dialog.getNumber();
x=Dialog.getNumber();
y=Dialog.getNumber();
xWidth=Dialog.getNumber();
yWidth=Dialog.getNumber();
list = getFileList(Idir);
if (getVersion>="1.40e")
 setOption("display labels", true);
setBatchMode(true);
for (i=0; i<list.length; i++){
 showProgress(i, list.length);
 processFile(Idir, Odir, list[i]);}
selectWindow("Results");
saveAs("Measurements", ""+Odir+"Results.txt");
selectWindow("Summary");
saveAs("Text", ""+Odir+"Summary.txt");
function processFile(Idir, Odir, filename)
{open(Idir + filename);
 makeRectangle(x, y, xWidth, yWidth);
 run("Crop");
 run("Subtract Background...", "rolling=50 disable");
//Tune rolling ball parameter based on background noise level and colony size
 setMinAndMax(1000, 49283);
 run("Apply LUT");
 setAutoThreshold("Default dark no-reset");
 run("Convert to Mask");
 run("Watershed");
 run("Analyze Particles...", "size=min_size-max_size circularity=min_circ-max_circ show=Overlay Mask display exclude summarize add composite");
 Opath = Odir + filename;
 saveAs("jpeg", Opath);
 close();}
Dialog.create("Validate")
Dialog.addMessage("Check output folder for image overlays, results, and summary")
Dialog.show();}
```

Normalization and Data Processing Methods for Figure 6

High CFU variability from non-optimized electroporation conditions and liquid handling effects necessitated duplicate E-Plate experiments to distinguish true parameter effects from technical artifacts. Normalization between plates was required to account for

systematic differences in absolute CFU counts, while data exclusion removed wells that might otherwise obscure genuine parameter–response relationships. The following section details our data processing procedures, including criteria for data exclusion, normalization methods, and a proportional ratio-based estimation method to complete our parameter space visualization, where one condition was missing from an E-Plate replicate due to a dispensing error.

Data Exclusion Criteria

Technical Exclusions

1. Sample Preparation Error:
   - One E-Plate replicate experienced a partial well-filling error during dispensing. This affected all wells in the 225 V, 0.16 ng/µl condition, and 13 consecutive wells in the 180 V, 0.16 ng/µl condition. These wells produced artificially reduced CFU counts and were excluded from analysis.

2. Electroporation Process Issues:
   - One well in the 275 V, 0.02 ng/µl condition was excluded as it failed to receive an electroporation pulse due to a solid-state relay malfunction, resulting in 0 CFU.
   - One well in the 225V, 0.02 ng/µl condition exhibited electrical arcing during electroporation and was excluded despite producing CFU.

Statistical Exclusions

The interquartile range (IQR) method was applied to detect and remove outliers from both E-Plate replicates, after the technical exclusions described above.

The IQR method identifies outliers based on the statistical dispersion of the dataset:

1. Calculation of Quartiles: For each experimental condition, we determined:
   - First quartile (Q1): the 25th percentile of the data;
   - Third quartile (Q3): the 75th percentile of the data;
   - Interquartile range (IQR): Q3 - Q1.

2. Determination of Outlier Boundaries:
   - Lower bound = Q1 - (1.5 × IQR);
   - Upper bound = Q3 + (1.5 × IQR).

An IQR multiplier or fence factor of 1.5 was selected as the standard value used in the IQR method for outlier detection, providing a balance between retaining natural variability and excluding genuine anomalies.

3. Outlier Identification: Data points falling below the lower bound or above the upper bound were classified as outliers and excluded.

This procedure identified 23 total outliers across both E-Plate replicates (13 in one replicate and 10 in the other). Among these outliers, 14 were high performers (above the upper bound) and 9 were low performers (below the lower bound).

Data Normalization and Integration

Coefficient of Variation Calculation

For each experimental condition, a unique normalization factor was calculated:

1. Condition-Specific Normalization Factor = Mean CFU (condition in E-Plate 1) / Mean CFU (same condition in E-Plate 2).

2. Individual CFU values from E-Plate replicate 2 were then adjusted by multiplying each value by its corresponding condition-specific normalization factor.

3. The normalized datasets were combined for subsequent coefficient of variation (CV) calculations across conditions.

Relative Efficiency Calculation

1. Within each E-Plate replicate, individual CFU values were expressed relative to the maximum CFU value observed on that plate:

Relative CFU = Individual CFU / Maximum CFU on respective E-Plate

2. For each condition, the average relative efficiency was calculated.

3. Finally, all condition averages were normalized to the highest-performing average:

Normalized Relative Efficiency = Average Relative Efficiency (condition) / Maximum Average Relative Efficiency (across all conditions)

Proportional Ratio-Based Estimation for Missing Condition

Since all CFU data on one E-Plate replicate for the 225 V, 0.16 ng/µl condition was excluded due to a dispensing error, we needed to estimate values for this condition to complete our analysis. We employed a proportional ratio-based estimation approach rather than standard interpolation. This method was selected due to the non-linear voltage–response relationships and high variability in our results. This approach leverages consistent proportional trends observed across multiple conditions, incorporating more experimental data points than interpolation while preserving the characteristic voltage-dependent patterns observed in both transformation efficiency and variability metrics.

To estimate the CV for the 225 V, 0.16 ng/µl condition:

1. For the next two lower plasmid concentrations (0.04 and 0.08 ng/µl), we calculated:
    - The CV difference between 180 V and 225 V conditions;
    - The CV difference between 180 V and 315 V conditions;
    - The ratio of these differences (180 V - 225 V) ÷ (180 V - 315 V).

2. The average ratio across both concentrations was determined, representing the proportional relationship between voltage steps.

3. For the 0.16 ng/μl concentration:
   - We calculated the CV difference between 180 V and 315 V conditions;
   - This difference was multiplied by the average ratio determined in Step 2;
   - The resulting value was subtracted from the CV of the 180 V, 0.16 ng/μl condition to obtain our estimated CV value for the missing 225 V, 0.16 ng/μl condition.

This approach leverages the voltage–response relationships observed at other concentrations to estimate the missing data point while maintaining the proportional trends observed in the dataset.

A similar approach was used to estimate the relative efficiency for this condition.

LISA Cluster Analysis Methodology

To identify and quantify spatial patterns in CFU across the E-Plate, we performed a Local Indicators of Spatial Association (LISA) cluster analysis. This analysis allowed us to establish the statistical significance of spatial clustering of CFU in the E-Plate, rule out the E-Plate and HTME as significant factors contributing to these clusters, and provide evidence that the observed spatial patterns likely result from liquid handling effects.

Neighborhood Definition

Each well in the E-Plate was assigned spatial coordinates based on its row and column position. For each well, we defined its "neighborhood" as all immediately adjacent wells (including diagonals).

<u>Local and Global Spatial Autocorrelation Calculation</u>

The analysis proceeded in sequential steps:

1. We standardized the raw CFU values by converting them to Z-scores:

$$Z = (CFU - \mu)/\sigma$$

where μ is the global mean CFU, and σ is the standard deviation.

The Z-score represents how many standard deviations a well's CFU differs from the mean CFU across the E-Plate.

2. For each well, we calculated the mean Z-score of its neighborhood:

$$\text{Neighborhood Z} = \frac{\sum_{(x_i,y_i)\in N(x_0,y_0)} Z_i - Z_0}{N(x_0,y_0) - 1}$$

where x and y are spatial coordinates, i is the well index, $N(x_0,y_0)$ is the neighborhood defined as all wells where $|x_i - x_0| \leq 1$ and $|y_i - y_0| \leq 1$, Z_i is the Z-score value for each neighboring well (x_i,y_i), and Z_0 is the Z-score of the current well being analyzed.

The symbol ∈ represents "is an element of" or "belongs to". In this formula, $(x_i,y_i)\in N(x_0,y_0)$ means “for all coordinate pairs (x_i,y_i) that are members of the neighborhood set $N(x_0,y_0)$”.

In practical terms, the formula finds the average Z-score of all wells that are immediately adjacent to a given well. It works by:

- Identifying all neighboring wells (those that are one position away);
- Adding up all the Z-scores from these neighboring wells;
- Subtracting the central well's own Z-score ;
- Dividing by the total number of neighbors (minus one to account for removing the central well).

3. Local Moran's I was calculated for each well as the product of its Z-score and its neighborhood Z-score:

$$\text{Local Moran's I} = Z \times \text{Neighborhood } Z$$

Local Moran's I quantifies whether each well’s CFU is part of a spatial cluster of wells with similar CFU or represents a spatial outlier. Local Moran’s I is positive when wells cluster with similar neighbors (both high or low CFU) and negative when wells differ from their surroundings.

4. Global Moran's I was calculated as the mean of all local Moran's I values.

Global Moran's I is a single statistic that measures the overall spatial autocorrelation of CFU across the entire E-Plate, indicating whether similar CFU values tend to cluster together (positive values), disperse (negative values), or distribute randomly (values near zero).

Cluster Classification

Each well was classified into one of four LISA cluster types based on the relationship between its Z-score and neighborhood Z-score:

- High–High: Wells with above-average CFU surrounded by wells with above-average CFU;
- Low–Low: Wells with below-average CFU surrounded by wells with below-average CFU;
- High–Low: Wells with above-average CFU surrounded by wells with below-average CFU;
- Low–High: Wells with below-average CFU surrounded by wells with above-average CFU.

LISA Cluster Maps

	1	2	3	4	5	6	7	8	9	10	11	12
A	Low Low	Low Low	Low Low	Low Low	Low High	High High	High High	High High	Low High	Low Low	High Low	Low Low
B	Low Low	High Low	Low Low	High Low	Low Low	Low Low	High Low	High High	High Low	Low Low	Low Low	Low Low
C	Low Low	Low Low	Low Low	High Low	Low Low	Low High	Low High	High High	Low High	Low Low	Low Low	Low Low
D	Low Low	Low Low	Low Low	Low Low	High Low	High Low	High Low	High Low	Low High	Low High	Low Low	Low Low
E	Low Low	Low Low	Low Low	Low Low	Low Low	Low Low	Low High	High High	High High	High High	High High	Low Low
F	Low Low	Low Low	Low Low	Low Low	Low Low	Low Low	High Low	Low Low	High High	High High	High High	High Low
G	Low Low	High Low	Low Low	High Low	Low Low	High Low	Low High	High High	Low High	Low High	High High	Low High
H	Low Low	Low Low	Low Low	Low Low	Low Low	Low Low	High Low	Low Low	High Low	High Low	High High	High Low
I	Low Low	Low Low	Low Low	Low Low	Low Low	Low Low	Low Low	High Low	Low High	Low Low	Low High	Low Low
J	Low Low	Low Low	Low Low	High Low	Low High	High Low	Low Low	High Low	High High	Low Low	High High	High Low
K	Low Low	High Low	Low High	High Low	Low High	Low Low	Low High	Low High	Low High	High High	Low High	High High
L	Low Low	Low Low	Low High	Low Low	Low High	Low Low	High High	High Low	High High	High High	High High	High High
M	Low Low	High Low	Low Low	High Low	Low High	High Low	Low High	High High	Low High	High High	Low High	High High
N	Low Low	Low Low	Low Low	High Low	High Low	High Low	Low High	High Low	Low High	High Low	Low Low	Low High
O	Low Low	Low Low	Low Low	Low Low	Low Low	Low Low	Low Low	Low Low	Low Low	Low Low	Low Low	Low Low
P	Low Low	Low Low	Low Low	Low Low	Low Low	Low Low	Low Low	Low Low	Low Low	High Low	Low Low	Low Low

	13	14	15	16	17	18	19	20	21	22	23	24
A	High Low	High Low	Low High	High Low	Low High	High High	Low High	High High	High High	High Low	Low High	High Low
B	Low Low	Low Low	Low Low	High Low	Low High	High Low	High High	High High	High High	High High	Low High	High Low
C	Low Low	Low Low	Low Low	Low Low	Low High	High High	Low High	High High	High High	High High	High High	High High
D	Low Low	Low Low	Low Low	Low Low	Low Low	High High	High High	High High	High High	High High	High High	High High
E	Low Low	High Low	Low Low	Low Low	Low High	High High	Low High	High High	Low High	High High	High High	High High
F	High Low	Low Low	Low Low	High Low	High Low	Low Low	High High	High High	High High	High High	High High	High High
G	Low High	High Low	Low High	Low Low	Low High	Low High	Low High	High High	Low High	High High	High High	High High
H	Low Low	Low Low	Low Low	High Low	Low Low	Low Low	High Low	High Low	High Low	High High	High High	High High
I	Low Low	Low Low	Low Low	High Low	Low Low	Low Low	Low High	Low High	Low High	Low High	High High	Low High
J	Low High	Low Low	Low Low	Low Low	Low Low	High Low	High High	High High	High High	Low High	High High	Low High
K	Low High	High Low	Low High	High Low	Low High	High High	Low High	High High	High High	High High	High High	High High
L	High High	High High	Low High	High High	High High	High High	High High	High High	High High	High High	High High	High High
M	High High	High High	High High	High High	High High	High High	High High	High High	High High	High High	High High	High High
N	High High	High High	High High	Low High	High Low	High High	High High	High High	High High	High High	High High	High High
O	Low High	Low High	Low High	Low High	Low Low	Low High	Low High	High High	Low High	High High	High High	High High
P	Low Low	High Low	High Low	Low Low	High Low	Low Low	Low Low	High Low	Low High	High High	High High	High High

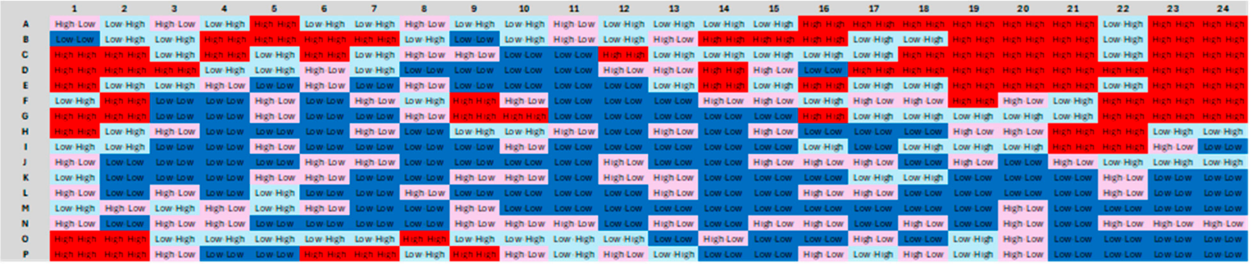

Supplementary Figure S1. LISA cluster maps comparing column-wise (top) and row-wise (bottom) dispensing methods. These maps visualize the spatial autocorrelation patterns of CFU across E-Plates analyzed in Section 3.2. Colors represent LISA cluster types: red (High–High), blue (Low–Low), pink (High–Low), and light blue (Low–High) as defined above.

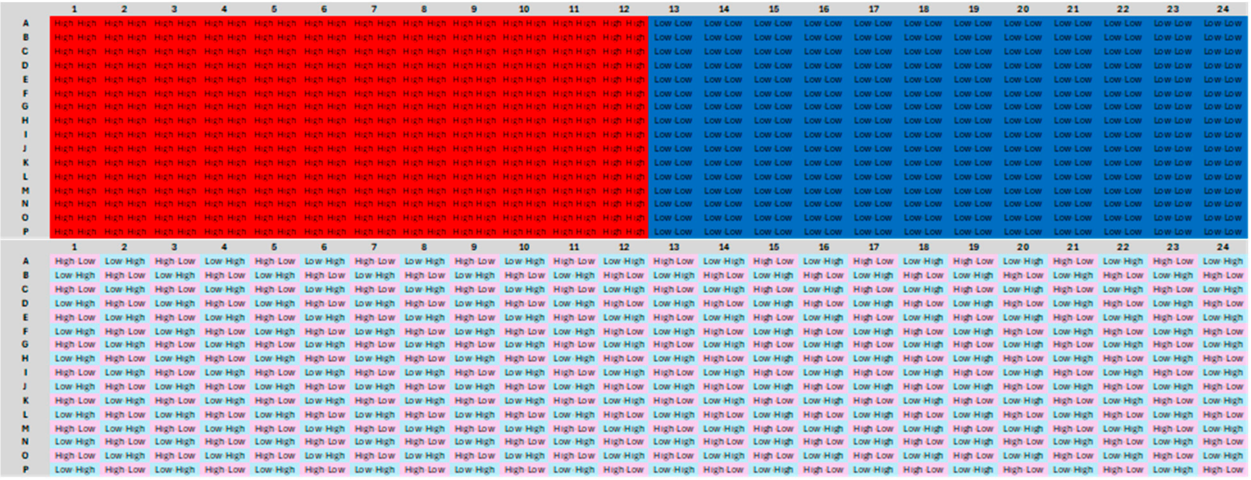

Supplementary Figure S2. Theoretical LISA cluster maps demonstrating extreme spatial autocorrelation patterns. These maps illustrate the visual representation of (top) maximum positive spatial autocorrelation (global Moran's I approaching +1), and (bottom) perfect negative spatial autocorrelation (global Moran's I = -1). These theoretical extremes provide reference points for interpreting the experimental cluster patterns observed in Figure S1, where our observed values (Moran's I = 0.28 and 0.15) indicate moderate positive spatial autocorrelation.

Pattern Similarity Calculation

The normalized pattern similarity between the LISA cluster maps in Figure S1 was calculated using a Simple Matching Coefficient (SMC), adjusted for random chance:

1. Simple Matching Coefficient (SMC): The proportion of wells that maintained the same cluster classification across both dispensing methods.
    - For each well position, a match was recorded (1) if the cluster type was identical in both methods, or a mismatch (0) if different;
    - SMC = sum of matches / total number of wells;
    - This raw similarity measure includes matches that would occur by random chance.

2. Normalization: Since there are four possible cluster categories, approximately 25% of wells would match by random chance alone, so we applied the following adjustment:

$$\text{Normalized Pattern Similarity} = (\text{SMC} - 0.25) / (1 - 0.25) \times 100\%$$

The resulting 2% normalized similarity indicates that, after accounting for random chance, there was almost no consistent pattern retention between the two dispensing methods. This near-complete reorganization of spatial clusters when only the dispensing pattern was altered provides strong evidence that the observed clustering cannot be attributed to inherent properties of the HTME system or E-Plate, as such device-related factors would produce consistent spatial patterns regardless of the dispensing order.
